# Performance Evaluation of Computer Vision Algorithms in a Programmable Logic Controller: An Industrial Case Study

**DOI:** 10.3390/s24030843

**Published:** 2024-01-28

**Authors:** Rodrigo Vieira, Dino Silva, Eliseu Ribeiro, Luís Perdigoto, Paulo Jorge Coelho

**Affiliations:** 1 School of Technology and Management, Polytechnic University of Leiria, 2411-901 Leiria, Portugaleliseu.ribeiro@ipleiria.pt (E.R.); luis.perdigoto@ipleiria.pt (L.P.); 2 Institute for Systems Engineering and Computers at Coimbra (INESC Coimbra), 3030-290 Coimbra, Portugal; 3 Institute of Systems and Robotics, University of Coimbra, 3030-290 Coimbra, Portugal

**Keywords:** programmable logic controllers, computer vision, OpenCV, performance benchmark

## Abstract

This work evaluates the use of a programmable logic controller (PLC) from Phoenix Contact’s PLCnext ecosystem as an image processing platform. PLCnext controllers provide the functions of “classical” industrial controllers, but they are based on the Linux operating system, also allowing for the use of software tools usually associated with computers. Visual processing applications in the Python programming language using the OpenCV library are implemented in the PLC using this feature. This research is focused on evaluating the use of this PLC as an image processing platform, particularly for industrial machine vision applications. The methodology is based on comparing the PLC’s performance against a computer using standard image processing algorithms. In addition, a demonstration application based on a real-world scenario for quality control by visual inspection is presented. It is concluded that despite significant limitations in processing power, the simultaneous use of the PLC as an industrial controller and image processing platform is feasible for applications of low complexity and undemanding cycle times, providing valuable insights and benchmarks for the scientific community interested in the convergence of industrial automation and computer vision technologies.

## 1. Introduction

Computer vision is an important area of industrial production systems, with numerous applications and use cases (e.g., quality control by automatic visual inspection, robot pick-and-place, and object sorting) [1,2]. Integrating computer vision with programmable logic controllers (PLCs) significantly advances industrial automation and control systems. This integration allows for real-time monitoring and precise control over complex manufacturing processes, enhancing the capabilities of PLCs beyond traditional control tasks. It increases efficiency, accuracy, and safety in applications like quality inspection, defect detection, and robotic guidance. This integration also supports Industry 4.0 technologies, fostering adaptable, efficient, and intelligent manufacturing environments [3,4]. Additionally, multi-sensor fusion approaches may enhance computer vision systems by integrating data from multiple sensors, overcoming individual sensor limitations. This technique improves system reliability and robustness, enhancing object detection, spatial understanding, etc. [5,6].

Performance evaluation in industrial settings involves thoroughly analyzing key factors like employee performance, machinery efficiency, production output, safety compliance, quality control, and cost efficiency. This process enhances productivity, maintains high standards, ensures safety compliance, and drives continuous improvement. Utilizing data-driven methodologies and proactive management, it maintains a competitive edge, optimizes operational efficiency, and creates a safe work environment [7]. Additionally, performance metrics in computer vision are crucial in industrial tasks like manufacturing, logistics, and quality inspection, as these metrics (for example, accuracy, processing speed, etc.) evaluate the effectiveness of computer vision systems in identifying defects, sorting products, and guiding robotic systems. These allow for minimizing errors and downtime, optimizing the efficiency and cost-effectiveness of processes. Quantifiable benchmarks allow industries to assess return on investment, facilitate continuous improvement, and ensure compliance with industry standards. The accurate evaluation and enhancement of computer vision systems are essential for maintaining a competitive advantage and operational excellence [8].

Integrating PLCs with computer vision systems involves various communication protocols. OPC-UA is renowned for its high security and interoperability, supporting complex data types and structures. Modbus TCP/IP is famous for its simplicity and reliability, making it suitable for real-time applications and supporting various hardware and software platforms. Ethernet/IP is advantageous for its high-speed data transfer capabilities, handling large volumes of data generated by computer vision systems. MQTT is popular in IoT applications due to its low bandwidth usage, making it ideal for remote or network-constrained environments. PROFINET is known for its real-time capabilities, support for high data rates, and minimizing latency, making it suitable for applications requiring precise synchronization between PLCs and vision systems. The protocol choice depends on the application’s requirements, such as data complexity, network conditions, and real-time processing needs [9,10].

Deep learning techniques are used in PLC-based computer vision systems to improve industrial automation capabilities. These techniques use Convolutional Neural Networks (CNNs) for image recognition and processing tasks, enabling real-time visual data analysis for object detection, classification, and localization. Transfer learning is often employed to leverage pre-trained models on large datasets, significantly reducing the time and data required for training specific to industrial applications. Recurrent neural networks, particularly Long Short-Term Memory (LSTM) networks, are used for tasks involving temporal dependencies in video data. These techniques transform PLCs into intelligent systems capable of complex visual tasks [11]. In the last decade, there has been a rapid development of techniques based on machine learning (ML), which have produced good results in applications that were challenging to “classical” algorithms [12].

ML-based techniques often require specific hardware (GPU/TPU–graphics/tensor processing units) for both the training phase of the models and the inference phase, while “classic” methods rely on more traditional computer architectures [11]. Independently of the type of methods used, in industrial applications, image processing is typically performed in a dedicated processing device (sometimes integrated with the camera’s internal hardware) [13]. The output from the image analysis is then communicated to other devices that are tasked with the system’s overall control, e.g., programmable logic controllers (PLCs).

The authors consider there to be a research gap because, to the best of our knowledge, there are no studies documented in the literature that use PLCs and computer vision systems in a single device. As such, the following research questions are considered: RQ1: What performance can be achieved on the system with this configuration? RQ2: What is the applicability of this type of system in a real industrial scenario?

This paper evaluates the feasibility of using a PLC to execute “classical” (i.e., non-ML) computer vision algorithms. In an industrial process where the PLC is already used as a controller for electromechanical systems, it would be advantageous to be able to use it simultaneously for visual processing without having to resort to an additional computing platform dedicated only to that function (be it a camera with an internal processing capacity or a computer).

A controller from the PLCnext family from Phoenix Contact [14] was used in this work. Although the PLCnext controllers can be used as a traditional PLC, they are based on a Linux operating system, allowing for the application of software tools typically associated with computers. The PLC under analysis is recommended for small/medium-range industrial control applications, so hardware limitations prevent it from having the same performance as a computer at the level of processing-intensive applications. This paper aims to quantify this difference in performance and investigate the limitations of using the PLC in image processing. A benchmark of several typical image processing methods is presented, testing the performance of the PLC against a computer to evaluate the feasibility of applying the PLC in a practical industrial scenario. A demonstration application is implemented considering a real industrial use case.

The main contributions of this paper are as follows:

- Quantitative evaluation of the performance of a PLC in image processing applications (to the best of our knowledge, no other work presents a similar study);
- Description of an application in a real industrial use case.

In Section 2, the related work presenting the relevant literature is given. The proposed benchmark algorithms and the hardware/setup for the experiments are depicted in Section 3. Section 4 details the implementation results for the benchmarking performance and the industrial case study. The results are discussed in Section 5, and finally, the conclusion is given in Section 6.

## 2. Related Work

The growing use of machine learning (ML) techniques in computer vision is also present in industry applications [15] and is one of the enabling technologies of the new paradigms of Industry 4.0 [3,4]. However, classic methods are still prevalent in many applications or combined with ML approaches [16,17]. Defect detection applications relevant to the case study presented in this paper are examples of how state-of-the-art methods rely on classical and ML techniques [13,18,19].

PLCs are commonly used to perform the first layer of machine control on the factory floor and are designed for real-time monitoring, analysis, and decision making. A typical system architecture relies on the PLC for actuator control and a dedicated platform for image processing (e.g., a computer or a smart camera) [20], with many examples of applications in manufacturing [21,22] and robotics [23,24]. Aydogmus and Talu [25] introduce a method for integrating vision-based measurement systems with PLCs. This system enhances accuracy and efficiency in industrial processes, improving product quality and reducing waste. However, its complexity and reliance on specialized hardware and software may lead to higher initial costs and operator training. Additionally, it may not be suitable in challenging environments like dust, smoke, or variable lighting conditions.

Lee et al. [26] discuss the creation of a machine vision module for programmable logic controllers, highlighting the need for a PLC-integrated machine vision system to reduce costs and improve implementation convenience. They proposed a new machine vision module based on PLCs. The system allows PLC users to implement vision systems through ladder programming, and the developed system was tested for performance through a sample inspection system. The paper also details the design of a ladder instruction set for interfacing with the vision library and discusses the applications and results of this new system.

Chauhan and Surgenor [27] explored three machine vision methods—Gaussian mixture models with blob analysis, optical flow, and running average—to identify faults in automated assembly machines. These methods are lauded for requiring less training, processing time, and faster fault detection compared to the spatiotemporal volume method. Notably, these approaches autonomously learn from initial video frames, showcasing the potential for enhanced operational efficiency in automated fault detection.

Darekar and Kulkarni [28] introduced a new industrial automation system that uses wireless PLCs and computer vision technology to improve gear sorting efficiency and accuracy. This system improves sorting speed and reduces errors due to advanced image processing capabilities. It offers a flexible, scalable solution but may require high-quality network connectivity for uninterrupted operations. The system’s unique combination of wireless communication and advanced image recognition could revolutionize manufacturing efficiency and reliability.

Koodtalang et al. [29] presented an automated inspection system that uses stereo vision and a programmable logic controller for the simultaneous shape classification and height measurement of manufactured parts. The system uses Raspberry Pi and OpenCV for computation and two standard webcams for stereo vision. It has a high accuracy in shape classification and precise height measurement. However, it faces potential complexity and cost issues.

Al Fahim et al.’s [30] paper discusses creating and implementing an automated cell using a programmable logic controller for industrial applications. It highlights the benefits of PLCs, such as improved efficiency, reliability, and flexibility. It covers technical aspects, hardware configuration, software programming, and component interfacing. It also discusses the practical implications of PLC technology, highlighting potential improvements in production speed, accuracy, and operational effectiveness. The paper concludes with case studies showcasing successful applications in real-world industrial settings.

Demanding applications, in terms of processing power, usually decentralize image analysis to the cloud or, more commonly, to computers on the factory edge [8,31,32]. However, for simple, low-processing applications, using low-power and cheap devices is of interest [33,34]. These devices can be off-the-shelf single-board computers [35] or custom-built embedded systems [2,35,36].

In this paper, we explore the possibility of using a PLC as a low-processing-power image analysis device, adding this function to the standard system controller role, without additional devices. Although some of the works previously discussed present some relevant topics, there are few examples of using a PLC as an image processing platform [37,38], and, to the best of our knowledge, no previous works have performed a quantitative performance analysis.

## 3. Materials and Methods

This section presents the primary components required for a performance analysis to be performed between the PLCnext device and a personal computer. All the hardware devices used in the experiments are described in Section 3.1. Several comparison tests were conducted to evaluate the feasibility of using the PLCnext controller as a platform for image processing, evaluating the execution times of several standard algorithms against a personal computer (benchmarking). A set of basic methods/techniques, regularly used as pre-processing or as an integral part of more complex algorithms, was considered.

The popular open-source library OpenCV [37,39], which implements some standard computer vision algorithms, was installed in the PLC (in its Linux component, as will be further described in Section 3.2.1). Various image processing methods were implemented in the PLC using the Python programming language, and the computer was used for comparison. The same set of Python applications was executed on both devices. Additionally, the setup for an industrial real case scenario is presented to address the system performance, as further described in Section 3.2.2.

### 3.1. Materials

#### 3.1.1. AXC F 2152 Controller

**Figure 1:**
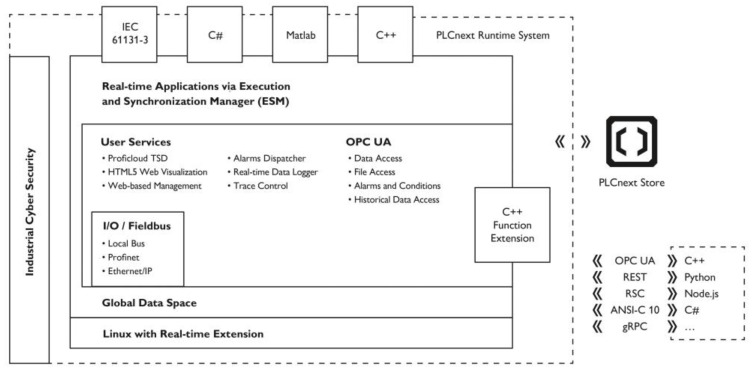
The general architecture of a PLCnext controller—source: Phoenix Contact (the authors thank Phoenix Contact for their permission to reproduce this image).

As mentioned, a Phoenix Contact industrial PLC, model AXC F 2152, which is part of the “PLCnext Technology” ecosystem, was used. Figure 1 presents the various components associated with the PLCnext architecture. The automaton operates based on a Linux kernel with real-time deterministic execution capability (using the patch preempt_rt [40]). 

A set of applications (PLCnext Runtime System) provides the user with a high-level interface with various functionalities, particularly the possibility of using traditional PLC programming languages, according to the IEC 61131-3 standard [41]. The controller can be used as a Linux platform or configured and programmed as a “classic” PLC (using the development environment provided by the manufacturer—PLCnext Engineer), where the Linux component is transparent to the user. Besides the “classic” PLC programming languages, code developed using Matlab/Simulink, C#, and C++ can be used in the controller’s real-time infrastructure. Downloading third-party applications and libraries is possible using an online service similar to an app store, called the “PLCnext Store” [42].

Various mechanisms allow communication between the “classic” component of the automaton and Linux applications (e.g., OPC-UA and REST-based protocols), making it possible to combine traditional PLC real-time control applications with a computer’s functionalities in a single device, where, for example, industrial IoT/IIoT (Industrial Internet of Things) applications can be executed, or an interface with cloud and edge computing [43].

The PLC is based on an ARM Cortex-A9 800 MHz Dual Core processor. Given its intended use as an industrial controller, it has hardware resources with considerably lower performance than what is usually available in platforms dedicated to intensive processing (computers).

#### 3.1.2. Personal Computer

In terms of hardware, the computer contained an Intel Core i5 processor, 3350 @ 3.10 GHz, 8 GB of RAM, and a Nvidia GeForce GT 630 graphics board. Similarly to the PLCnext controller, a Linux operating system (Ubuntu) and the OpenCV library were installed on the computer used for comparison, and the same Python applications were executed, disabling processing acceleration with the graphics processing unit in OpenCV.

#### 3.1.3. Camera Teledyne Dalsa

The Teledyne Dalsa Genie Nano G3-GM11-M1920 industrial camera [44] was used to obtain monochromatic images up to a maximum resolution of 1936 × 1216 pixels. The main characteristics are presented in Table 1. 

Communication between the PLC and the camera was achieved using the “GigE-V Framework 2.1” library, made available by the manufacturer for Linux systems [45]. The Wrapper “PyGigE-V” [46], which allows for the use of the library in programs developed in the Python programming language, was also used. This library can configure various camera parameters, send capture commands, and receive images.

### 3.2. Methods

In this subsection, we present some simple and classic computer vision algorithms used to compare the performance of the PLC compared to a personal computer. This performance is evaluated to verify its application as proof of concept in a real yet simplified case of industrial quality control.

#### 3.2.1. Image Processing Algorithms

As described, the methods were obtained from the open-source library OpenCV in Python and ran in a Linux environment, both on the PLCnext and a personal computer. Monochrome images with a resolution of 1920 × 1080 pixels gathered from the previously described Dalsa Genie Nano camera were used as the input to the algorithms. The set of tested algorithms is listed and briefly described in Table 2.

The methods and operations listed in Table 2 can be grouped into categories based on their functionality within image processing. The first category includes basic image thresholding methods such as simple threshold, Threshold Otsu, and Adaptive Threshold, which are used to binarize images, converting images from intensity values into binary form. The second category comprises operations that modify binary image structure, like Invert for pixel-by-pixel flipping, Erode for narrowing regions in binary images, and Dilate for expanding regions in binary images. The third category is an image resize operation to provide new dimensions to an existing frame. The fourth category encompasses edge and contour detection methods, including Canny Edge Detector and Find Contours. Some diffusion filters, namely the well-known Gaussian Blur, are typically applied to soften the images for better contour identification. The fifth category relates to image analysis and matching techniques, which consist of template matching for finding correspondence with a reference image and keypoint detectors, like ORB, to deal with the detection of characteristic points and establishing correspondences between images, focusing on the detection and description of feature points.

#### 3.2.2. Quality Control Sequence Description

**Figure 2:**
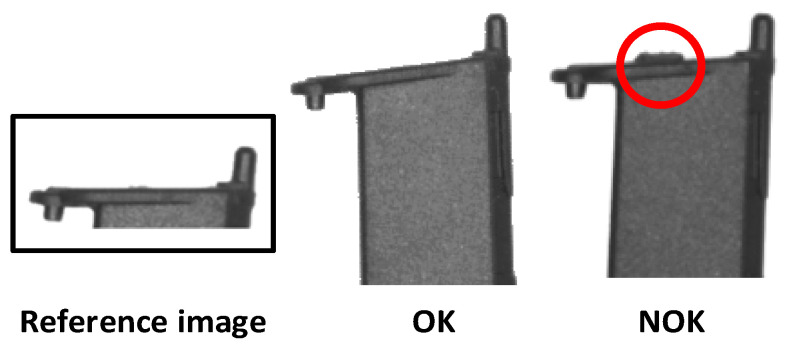
Reference image and examples of OK and NOK parts.

The difficulty of quality control arises from human errors in checking the quality of injected plastic parts, which leads to many customer complaints due to the delivery of non-conforming parts, according to the study’s partner company. Quality control is applied at one extremity of the part. The objective is to determine whether the image of each piece matches a previously defined reference image. Figure 2 depicts the reference image, an OK (conforming) part, and a part that, due to excess material (an example is depicted in the red circle), should be classified as non-conforming—NOK.

**Figure 3:**
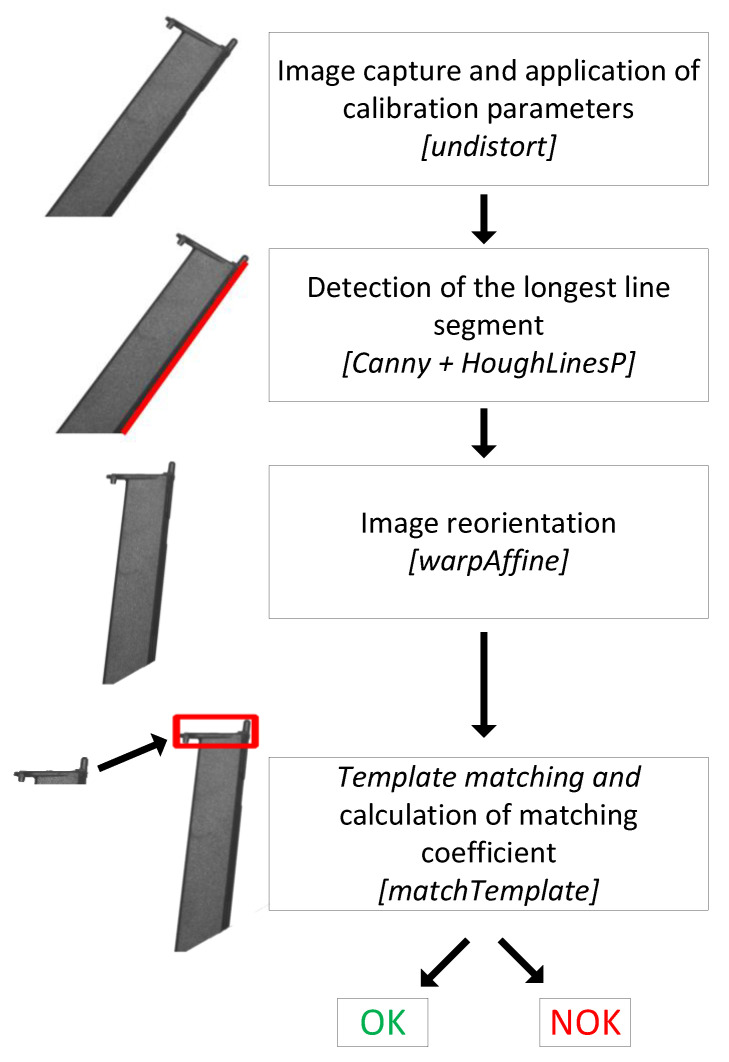
Image processing algorithm, indicating the main OpenCV functions used in the implementation.

The image processing algorithm is summarized in Figure 3. The camera is pre-calibrated by obtaining a set of images of a checkerboard pattern of known dimensions. This procedure is performed only once before the system goes into operation. Calibration minimizes image distortions caused by imperfections in the camera’s lens and sensor and is a standard procedure fully supported in the OpenCV library.

In the first step of the algorithm, the image is captured, and the distortions are corrected by applying the parameters obtained in the initial calibration.

It was considered that the part could be positioned in any orientation over a horizontal plane. Because of this, a rotation is applied to the image so that the orientation is the same as the reference image. The longest edge in the object is identified, and the image is rotated so that this edge has a predetermined angle (so that the top of the part is horizontal).

Finally, the reference image is searched in the test image, and the template matching technique is used to obtain the location and a matching coefficient (value range from 0% to 100%). By extensive experimentation, applying the algorithm to a set of test images with OK and NOK parts, it was defined that the parts are classified as NOK whenever the matching coefficient is less than 97%.

### 3.3. Case study Scenario Description

**Figure 4:**
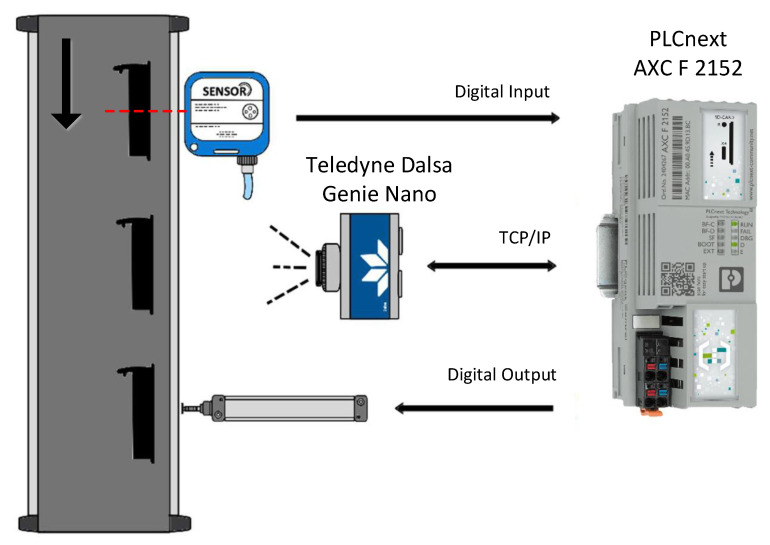
Scenario considered for the demonstration application.

Figure 4 schematizes the system under consideration. A conveyor transports the parts to be analyzed. A photoelectric sensor detects the arrival of a new part. A camera captures an image of the part. Depending on the result of the image processing, the part is classified as conforming (OK) or non-conforming (NOK). Non-conforming parts are removed from the conveyor via a pneumatic actuator.

The PLCnext controller is connected to the photocell and actuator via digital input/output (I/O) signals. The connection to the camera is made using TCP/IP communication. To represent a typical industrial application, the configuration and programming of the sensor and actuator signals are performed in the “classic” component of the automaton, and the interface with the camera and the image processing is constructed in the “Linux” component (thus eliminating the need to use an external computer).

A simple program was established using standard PLC ladder logic language (IEC 61131-3), which sends a signal to the “Linux” component of the controller when there is a new part detected, waits for the result of image processing, and activates the actuator when the part is classified as NOK. For simplicity, it was considered that the conveyor has a constant speed, and the alignment of the parts with the pneumatic actuator is achieved through a timer.

As in the performance tests described above, the image processing application was developed in Python using the OpenCV library. Communication between the Python program and the program variables in the Ladder programming language of the “classical” controller was made using a REST protocol provided by the PLC’s operating system [47,48]. Variables in the controller’s global data space (see Figure 1), where the process variables of the “classic PLC” component are stored, can be configured to be available through a REST-based protocol. A REST client was used in the Linux component to read the sensor signal and write the OK/NOK response back to the “classic” PLC component.

## 4. Results

To delve into a comprehensive analysis of the performance benchmarks of the selected algorithms, we focus first on the comparative efficiency and effectiveness of these algorithms when operated on two distinct devices, each with unique specifications and capacities. Secondly, we apply some of these algorithms in a practical case study situation, offering valuable insights into their real-world applicability and performance under typical operational conditions. This section presents a detailed overview of the algorithms’ capabilities, highlighting their strengths and limitations, thereby offering a holistic understanding of their practical utility in the field.

### 4.1. Benchmark Results for the PLC and PC

**Figure 5:**
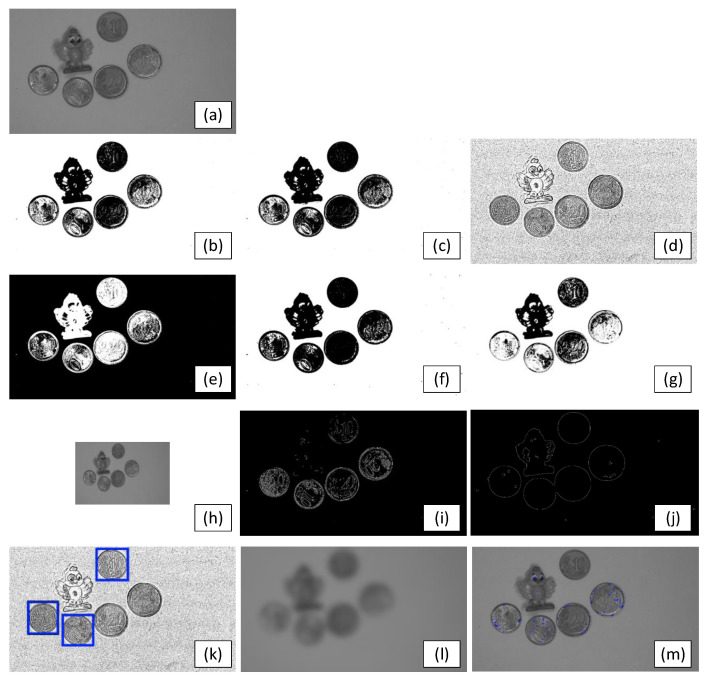
Test images. (**a**) Original image. (**b**–**m**) Results produced by each of the algorithms listed in Table 2 (in the same order).

The results of evaluating the methods described in Section 3.2.1 are presented in Figure 5, which provides the individual visual achievements.

**Figure 6:**
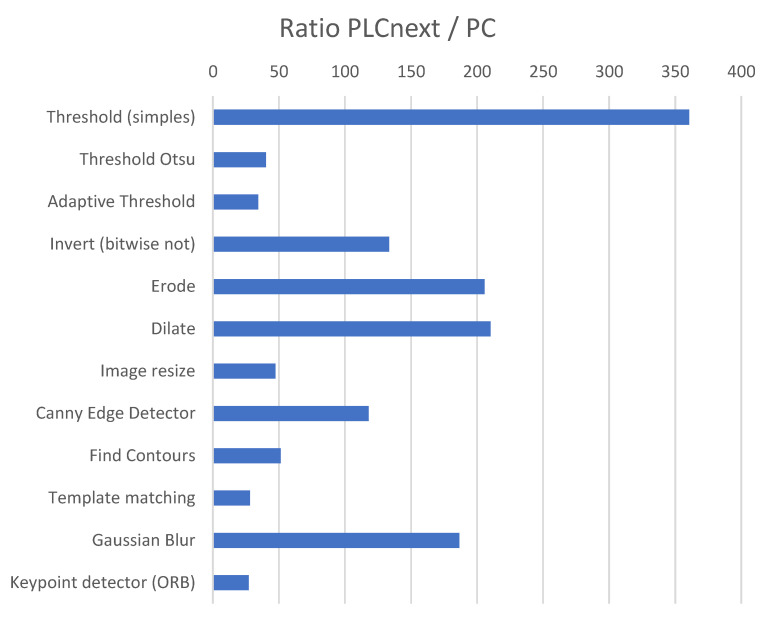
Execution time ratio between PLCnext and PC.

Each algorithm was executed 1000 times (both in the PLC and the computer), and the arithmetic average of the execution times was calculated. Table 3 shows the obtained times. Additionally, Figure 6 shows the ratio between the average execution times of the PLC and the computer.

The difference in performance between the two platforms is evident. The type of use for which the PLC is designed does not require intensive processing (priority is given to other factors such as reliability and robustness); therefore, its use as an image processing platform has many limitations. Still, considering the absolute times presented in Table 3, a set of less demanding algorithms was run under 100 ms intervals, which may be feasible for simple applications with not very demanding cycle times. 

The template matching method had the longest execution time, exceeding 4 s. As an important method in quality control applications, it was used as an integral part of the demonstration application described below to evaluate performance in a realistic scenario of a vision-based quality control process.

### 4.2. Results Obtained in the Case Study

**Figure 7:**
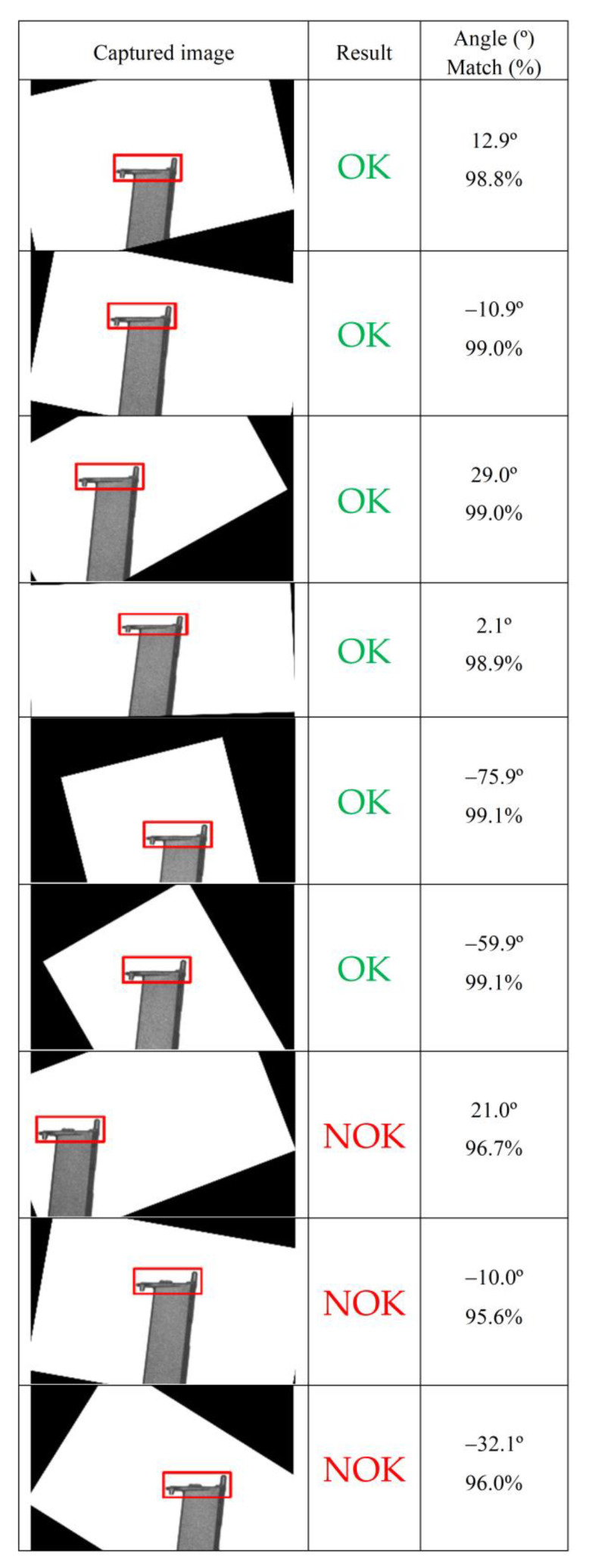
Some examples of the detection and conformity results obtained.

Following the setup shown in Figure 5, some conformity tests were carried out on real plastic parts. Figure 7 shows some examples of the experimental results. It presents the value of the rotation angle of the image, the image of the part already reoriented according to that angle (so that it has a horizontal top), the value of the matching coefficient, and the final assigned classification (OK/NOK). In the NOK images, it is possible to observe the excess material present in the part that led to its rejection.

For the demonstration application, the camera was set to a resolution of 968 × 608 pixels. This resolution was chosen through experimental tests because it produced good results while saving some processing time. Still, the average execution time of the image processing algorithm was about 8 s per piece.

Despite the success in correctly classifying the parts, the execution time is high compared to the usual performance of image-processing-dedicated hardware. However, this result demonstrates that the use of the PLC is feasible in real visual inspection applications, whose demands in terms of cycle times are low.

## 5. Discussion

This work performs a benchmark test of several basic methodologies or algorithms used in computer vision in a PLC. It performs a comparison to a standard computational system and provides an evaluation of the execution times. It also allows for inferring the order of magnitude of the execution times necessary to build any real industrial application, for example, the analysis of defects in parts. 

From the benchmark test, it can be seen that for the simpler methods, the execution times approach 100 ms, which can be considered acceptable for real-time applications. More complex algorithms require an order of magnitude higher (until 40 times higher in our tests). The template matching method, for example, requires over 4 s. In the real scenario use case (that used template matching), it was shown that the cycle time per part was 8 s, which may be on the verge of feasibility for demanding applications.

Answering “RQ1: What performance can be achieved on the system with this configuration?”, the authors claim that it is not fair to compare directly with other scientific studies since no works are entirely similar to the one presented in this paper. The studies reported in the literature typically present computer vision systems interconnected with computers or other IoT/IIoT devices. This difference is vital given that one of the advantages of this approach is the ability to have all processing integrated into a single device (PLC), which is, by construction, more robust for the industrial environment when compared to IoT/IIoT devices. Still, it is not a device dedicated to intensive processing tasks (like image processing). There is, therefore, a need to obtain a trade-off between expected performance, simplicity in implementation, and robustness of the equipment itself. With the increase in the processing capacity of these devices and the tendency for PLCs to have operating systems that are more similar to those of computers (for example, Linux), the performance of PLC devices may improve. It should also be noted that many of these PLC devices already have their own security protocols, often proprietary, and some studies are already exploring these protocols in this type of device [49,50]. Industrial computer vision technology faces security and privacy concerns due to unauthorized access to sensitive data and surveillance. It leads to developing secure, privacy-conscious systems while balancing efficiency and innovation.

Regarding the industrial demonstration and answering “RQ2: What is the applicability of this type of system in a real industrial scenario?”, it appears that for applications with low demands on execution times, these devices can provide acceptable performance for the computer vision component, although they are significantly inferior when compared to a dedicated system.

The future of systems integrating PLCs with computer vision is poised for transformative advancements. We are likely to see these systems become increasingly autonomous, using machine learning algorithms to enhance their decision-making capabilities. As computer vision technology evolves, these integrated systems will offer more precise and versatile image analysis, enabling them to handle complex tasks more efficiently. The integration will facilitate real-time monitoring and predictive maintenance, predicting failures before they occur, and reducing downtime. There is also a potential shift towards edge computing, where data processing occurs on or near the PLC, leading to faster response times and reduced reliance on cloud-based systems. This could be particularly beneficial in remote or latency-sensitive applications.

Future research in integrating computer vision with PLCs also encompasses several pivotal areas. A primary focus is algorithm optimization, which involves tailoring computer vision algorithms to function efficiently within PLC systems while preserving their effectiveness in industrial settings. Concurrently, understanding the hardware constraints of PLCs is crucial, mainly how their inherent limitations influence the implementation of complex computer vision algorithms. Additionally, real-time processing is a significant aspect, examining the ability of PLCs to handle demanding, real-time image processing tasks in industrial environments. Another vital area is robustness and reliability, questioning the dependability of PLC-based computer vision systems in diverse industrial environments, which often vary in terms of exposure to elements and operational demands. When proven advantageous, the scalability of these systems is also important, especially in determining how well PLC-based computer vision solutions can adapt to different sizes and types of industrial applications, ensuring that they can meet varying demands without significant overhauls. This will lead to a cost–benefit analysis, where industries must assess if merging computer vision with PLCs is more cost-effective than utilizing separate, dedicated image processing units, including the integration challenges that need a thorough examination, focusing on the main hurdles encountered when embedding computer vision algorithms into existing PLC systems. This task might require changes in both hardware and software paradigms.

## 6. Conclusions

This work aimed to evaluate the feasibility of adding the task of image processing to the usual functions of monitoring the signals of sensors/actuators and performing system control of an industrial PLC. The AXC F 2152 controller from Phoenix Contact’s PLCnext range was used, and its capability as a Linux platform was explored for implementing computer vision applications.

A quantitative performance study was presented, compared to a personal computer, in the execution of some traditional computer vision algorithms. A demonstration application was also implemented, considering a vision-based quality inspection scenario.

Since intensive processing is not a requirement of the tasks for which the PLC is designed, the performance limitations in execution times are considerable. Even so, it has been demonstrated that this type of application is feasible and that image processing algorithms can be developed using standard high-level tools in other computing platforms (Python language, OpenCV library).

The PLC considered in this work can accumulate this function in industrial applications requiring simple image processing and low cycle time demands. Depending on the processing algorithm required, the benchmark results presented in this paper may be used to establish an initial estimate of the achievable performance.

The convergence of IT (Information Technology), focused on communication and data processing, and OT (Operational Technology), focused on low-level industrial systems control, is one of the trends of the Industry 4.0 revolution. The device used in this paper follows this trend by combining the functions of a traditional PLC with a platform that enables IoT and other computer-specific tasks. As these types of devices evolve, integrating computationally demanding image processing applications becomes feasible in terms of hardware and computing power.

## Figures and Tables

**Table 1:** The main characteristics of the Camera Genie Nano M1920 Mono—source: [44].

Property	Description
Sensor	Sony IMX249
Pixel Size	5.86 µm
Signal	Monochrome
Resolution	1936 × 1216 pixels
Total Pixels	2.35 Mpixels
Bit depth	8-bit, 12-bit
Dynamic range	75 dB
Interface speed	1 Gbps
Shutter type	Global shutter
Lens options	C-Mount, CS-Mount
Power requirements	Power over Ethernet (PoE) or 10–36 VDC
Operating temperature	−20 °C–65 °C
Dimensions (W × H × L)	44 × 29 × 21 mm
Mass	46 g

**Table 2:** Methods/operations used in benchmarking.

Method/Operation	Description
Threshold (simple)	Binarization with a fixed threshold
Threshold Otsu	Binarization with automatic threshold
Adaptive Threshold	Adaptive binarization by regions
Invert (bitwise not)	Pixel-by-pixel flipping
Erode	Erosion of regions in binary images
Dilate	Dilation of regions in Binary images
Image resize	Image resizing (new dimension: 800 × 600 pixels)
Canny Edge Detector	Contour detection
Find Contours	Contour detection
Template matching	Correspondence against a reference image
Gaussian Blur	Diffusion filter
Keypoint detector (ORB)	Detection of characteristic points and establishment of correspondences between images (only the detection and description of feature points functionality was used)

**Table 3:** Average execution times of the algorithms.

Method/Operation	PLCnext—Execution Time (ms)	PC—Execution Time (ms)
Threshold (simple)	32.7481	0.0908
Threshold Otsu	50.1426	1.2476
Adaptive Threshold	151.8254	4.4210
Invert (bitwise not)	23.1659	0.1735
Erode	97.6874	0.4749
Dilate	102.0167	0.4851
Image resize	42.0595	0.8883
Canny Edge Detector	405.0961	3.4341
Find Contours	110.5418	2.1554
Template matching	4403.2212	157.4470
Gaussian Blur	101.3707	0.5431
Keypoint detector (ORB)	1684.6446	62.0268

## Data Availability

Data will be available by request to the corresponding author.

## References

[1] Torras C. (1992). Computer Vision: Theory and Industrial Applications.

[2] Kotseruba I., Papagelis M., Tsotsos J.K. (2021). Industry and Academic Research in Computer Vision. arXiv.

[3] Lemstra M.A.M.S., De Mesquita M.A. (2023). Industry 4.0: A Tertiary Literature Review. Technol. Forecast. Soc. Chang..

[4] Javaid M., Haleem A., Singh R.P., Rab S., Suman R. (2022). Exploring Impact and Features of Machine Vision for Progressive Industry 4.0 Culture. Sens. Int..

[5] Aggarwal J.K. (2013). Multisensor Fusion for Computer Vision.

[6] Blum R.S., Liu Z. (2018). Multi-Sensor Image Fusion and Its Applications.

[7] Lwakatare L.E., Raj A., Crnkovic I., Bosch J., Olsson H.H. (2020). Large-Scale Machine Learning Systems in Real-World Industrial Settings: A Review of Challenges and Solutions. Inf. Softw. Technol..

[8] Lema D.G., Usamentiaga R., García D.F. (2024). Quantitative Comparison and Performance Evaluation of Deep Learning-Based Object Detection Models on Edge Computing Devices. Integration.

[9] Wilamowski B.M., Irwin J.D. (2016). Industrial Communication Systems.

[10] Bartoň M., Budjač R., Tanuška P., Schreiber P., Horák T. (2021). Industry Communication Based on TCP/IP Protocol. Res. Pap. Fac. Mater. Sci. Technol. Slovak Univ. Technol..

[11] Mahony N.O., Campbell S., Carvalho A., Harapanahalli S., Velasco-Hernandez G., Krpalkova L., Riordan D., Walsh J. (2020). Deep Learning vs. Traditional Computer Vision. Advances in Computer Vision: Proceedings of the 2019 Computer Vision Conference (CVC), Las Vegas, NV, USA, 25–26 April 2019.

[12] Raut R., Krit S., Chatterjee P. (2022). Machine Vision for Industry 4.0: Applications and Case Studies.

[13] Ren Z., Fang F., Yan N., Wu Y. (2022). State of the Art in Defect Detection Based on Machine Vision. Int. J. Precis. Eng. Manuf.-Green Tech..

[14] PLCnext Technology|Phoenix Contact. https://www.phoenixcontact.com/pt-pt/industrias/plcnext-technology.

[15] Jan Z., Ahamed F., Mayer W., Patel N., Grossmann G., Stumptner M., Kuusk A. (2023). Artificial Intelligence for Industry 4.0: Systematic Review of Applications, Challenges, and Opportunities. Expert Syst. Appl..

[16] Zhou L., Zhang L., Konz N. (2023). Computer Vision Techniques in Manufacturing. IEEE Trans. Syst. Man Cybern Syst..

[17] Tang Y., Sun K., Zhao D., Lu Y., Jiang J., Chen H. (2022). Industrial Defect Detection Through Computer Vision: A Survey. Proceedings of the 2022 7th IEEE International Conference on Data Science in Cyberspace (DSC).

[18] Wahab Hashmi A., Singh Mali H., Meena A., Farukh Hashmi M., Dhanraj Bokde N. (2023). Surface Characteristics Measurement Using Computer Vision: A Review. Comput. Model. Eng. Sci..

[19] Benbarrad T., Salhaoui M., Kenitar S.B., Arioua M. (2021). Intelligent Machine Vision Model for Defective Product Inspection Based on Machine Learning. JSAN.

[20] Moru D.K., Agholor D., Imouokhome F.A. (2021). Machine Vision and Metrology Systems: An Overview. Int. J. Data Sci..

[21] Aboah Boateng E., Bruce J.W. (2022). Unsupervised Machine Learning Techniques for Detecting PLC Process Control Anomalies. JCP.

[22] Mo F., Ugarte Querejeta M., Hellewell J., Rehman H.U., Illarramendi Rezabal M., Chaplin J.C., Sanderson D., Ratchev S. (2023). PLC Orchestration Automation to Enhance Human–Machine Integration in Adaptive Manufacturing Systems. J. Manuf. Syst..

[23] Kuang Y. (2022). A Perspective of Intelligent Algorithms and Manipulator Control. Proceedings of the 2022 4th International Conference on Robotics, Intelligent Control and Artificial Intelligence.

[24] Ayten K.K. (2019). Real-Time Implementation of Image Based PLC Control for a Robotic Platform. Balk. J. Electr. Comput. Eng..

[25] Aydogmus O., Talu M.F. (2012). A Vision-Based Measurement Installation for Programmable Logic Controllers. Measurement.

[26] Lee S.-B., Park T.-H., Han K.-S. (2014). Development of Machine Vision System Based on PLC. J. Inst. Control Robot. Syst..

[27] Chauhan V., Surgenor B. (2015). A Comparative Study of Machine Vision Based Methods for Fault Detection in an Automated Assembly Machine. Procedia Manuf..

[28] Darekar Y., Kulkarni S., Merchant S.N., Warhade K., Adhikari D. (2021). Automatic Gear Sorting Using Wireless PLC Based on Computer Vision. Advances in Signal and Data Processing.

[29] Koodtalang W., Sangsuwan T., Noppakaow B. (2018). A Design of Automated Inspections of Both Shape and Height Simultaneously Based on Stereo Vision and Plc. Proceedings of the 2018 18th International Conference on Control, Automation and Systems (ICCAS).

[30] Al Fahim A., Rahman M.M., Hridoy M.W., Uddin K.R. (2023). Development of a PLC Based Automation Cell for Industry. J. Integr. Adv. Eng..

[31] Liu F., Tang J., Yang J., Wang H. (2023). Automated Industrial Crack Inspection System Based on Edge-Edge Collaboration of Multiple Cameras and Programmable Logic Controller. Proceedings of the 2023 IEEE International Symposium on Broadband Multimedia Systems and Broadcasting (BMSB).

[32] Murshed M.G.S., Murphy C., Hou D., Khan N., Ananthanarayanan G., Hussain F. (2022). Machine Learning at the Network Edge: A Survey. ACM Comput. Surv..

[33] Alyamkin S., Ardi M., Berg A.C., Brighton A., Chen B., Chen Y., Cheng H.-P., Fan Z., Feng C., Fu B. (2019). Low-Power Computer Vision: Status, Challenges, and Opportunities. IEEE J. Emerg. Sel. Top. Circuits Syst..

[34] Goel A., Tung C., Lu Y.-H., Thiruvathukal G.K. (2020). A Survey of Methods for Low-Power Deep Learning and Computer Vision. Proceedings of the 2020 IEEE 6th World Forum on Internet of Things (WF-IoT).

[35] Nair D., Pakdaman A., Plöger P.G. (2020). Performance Evaluation of Low-Cost Machine Vision Cameras for Image-Based Grasp Verification. arXiv.

[36] Meribout M., Baobaid A., Khaoua M.O., Tiwari V.K., Pena J.P. (2022). State of Art IoT and Edge Embedded Systems for Real-Time Machine Vision Applications. IEEE Access.

[37] OpenCV—Python, Red Light Detection on PLCnext. https://www.plcnext-community.net/makersblog/opencv-python-red-light-detection-on-plcnext/.

[38] Bradski G.R., Kaehler A. (2011). Learning OpenCV: Computer Vision with the OpenCV Library.

[39] PLCnext Technology|Camera and Vision. https://www.plcnext-community.net/forum/.

[40] Intro to Real-Time Linux for Embedded Developers—Linux Foundation. https://www.linuxfoundation.org/blog/blog/intro-to-real-time-linux-for-embedded-developers.

[41] IEC 61131-3:2013|IEC Webstore|Water Automation, Water Management, Smart City. https://webstore.iec.ch/publication/4552#additionalinfo.

[42] PLCnext Store|The Open Software Store for Automation. https://www.plcnextstore.com/eu/.

[43] Chalapathi G.S.S., Chamola V., Vaish A., Buyya R. (2019). Industrial Internet of Things (IIoT) Applications of Edge and Fog Computing: A Review and Future Directions. Fog/Edge Computing for Security, Privacy, and Applications.

[44] Genie Nano-1GigE|Teledyne DALSA. https://www.teledynedalsa.com/en/products/imaging/cameras/genie-nano-1gige/.

[45] GigE-V Framework for Linux|Teledyne DALSA. https://www.teledynedalsa.com/en/support/downloads-center/software-development-kits/132/.

[46] Cramer J. Jcramer/pyGigE-V. https://github.com/jcramer/pyGigE-V.

[47] REST Data Interface. https://www.plcnext-community.net/infocenter/rest_data_interface_introduction/.

[48] Skachkov O. AlexanderSkachkov/pyPLCn. https://github.com/AlexanderSkachkov/pyPLCn.

[49] Ackerman P. (2017). Industrial Cybersecurity: Efficiently Secure Critical Infrastructure Systems.

[50] Kayan H., Nunes M., Rana O., Burnap P., Perera C. (2022). Cybersecurity of Industrial Cyber-Physical Systems: A Review. ACM Comput. Surv..

[51] EduNet|Mais Um @ DEE—ESTG—IPLERIA. https://sites.ipleiria.pt/edunet/.

